# Assessment of *Bla*_TEM_, *Bla*_SHV_, and *Bla*_CTX-M_ genes of antibiotic resistance in Gram-negative bacilli causing urinary tract infections in Khartoum State: a cross-sectional study

**DOI:** 10.1186/s12879-024-09023-7

**Published:** 2024-01-29

**Authors:** Manal Ismail Abdalla Mohammedkheir, Elsheikh Mahgoub Gaafar, Eltayeb GareebAlla Eltayeb AbdAlla

**Affiliations:** 1 Sudan International University, Khartoum, Sudan; 2 Faculty of Medicine, Microbiology Department, Alzaiem Alazhari University, Khartoum North, Sudan; 3 University of Khartoum, Khartoum, Sudan; 4 University of London, London, UK

**Keywords:** Urinary tract infections, Antibiotic resistance, Resistance genes

## Abstract

**Background:**

Gram-negative bacilli are the most common etiological agents responsible for urinary tract infections. The prevalence of antibiotic resistance in Gram-negative bacilli is increasing at a rapid pace globally, which is constraining the available choices for UTI treatment. The objectives of this study are to identify the most common causal organisms of urinary tract infections (UTIs), and to determine their drug resistance patterns.

**Materials and methods:**

This was a cross-sectional hospital-based study conducted at El-Amal Hospital, Bahri Teaching Hospital, and Al-Baraha Hospital, Khartoum State, from March to October 2022. Urine samples from patients suspected to have UTI were collected, and patients with confirmed UTI by laboratory investigations and yielded culture growth were enrolled. Antibiotic sensitivity testing and PCR testing of the *bla*_TEM_, *bla*_SHV_, and *bla*_CTX-M_ genes were done.

**Results:**

This study included 50 patients with UTI out of 229 suspected patients (21.8%). The most prominent group of patients was older than 60 years (40%); the majority were females (70%). *Escherichia coli* was the most prevalent isolated organism (50%), followed by *Klebsiella oxytoca* (24%), *Klebsiella pneumoniae* (20%), *Pseudomonas aeruginosa* (4%), and *Citrobacter freundii* (2%). A small percentage of organisms were resistant to colistin (17%). However, 77% were resistant to amikacin, 97.6% to cefotaxime, 96.8% to ceftazidime, 97.6% to ceftriaxone, 96.8% to cefixime, 87.6% to ciprofloxacin, 88.4% to gentamycin, 62% to imipenem, 67.6% to meropenem, 87.6% to norfloxacin, and 95.6% to trimethoprim. The overall resistance of isolated gram-negative organisms was 81%. The most prevalent gene for the resistance was *bla*_TEM_ (100%), followed by *bla*_CTX-M_ (94%), and then *bla*_SHV_ (84%).

**Conclusion:**

*Escherichia coli* and *Klebsiella species* were the most commonly isolated uropathogens in this study, and the majority were highly resistant to most of the antimicrobial agents tested. Resistance genes *bla*_TEM_, *bla*_CTX-M_, and *bla*_SHV_ are very common in uropathogens.

**Supplementary Information:**

The online version contains supplementary material available at 10.1186/s12879-024-09023-7.

## Introduction

Urinary tract infections (UTIs) are among the most common bacterial infections [1]. It affects almost 150 million people each year worldwide [2]. Urinary tract infections are more commonly caused by Gram-negative bacteria such as “Enterobacterales,” which cause both community-acquired and hospital-acquired UTIs, which is a growing concern due to limited therapeutic options [3]. Patients suffering from symptomatic UTIs (fever, pain during urination, lethargy) are usually treated with antibiotics, resulting in multidrug resistance [4].

Gram-negative bacilli produce antibiotic-inactivating enzymes and non-enzymatic processes, which are the causes of antimicrobial resistance [5]. They may be expressed intrinsically by a given species (chromosomal genes) or acquired by a subset of strains as a result of: (1) mutations in chromosomal genes, resulting in increased expression of intrinsic resistance mechanisms, permeability alterations by loss of outer membrane porins, or target modifications. (2) Horizontal transfer of mobile genetic elements (MGEs) with resistance genes, particularly those generating plasmid-encoded beta-lactamases, aminoglycoside-modifying enzymes (AMEs), or non-enzymatic techniques [6].

The most important mechanism of resistance in Gram-negative bacilli is based on the production of β-lactamases, enzymatic proteins that hydrolyze β-lactam rings [7]. These genes are either penicillinases (TEM-1/2 and SHV-1) that break down penicillins and first- and second-generation cephalosporins or extended-spectrum-β-lactamases (ESBL) (TEM-3, SHV-2, and CTX-M) that break down the third generation of cephalosporins [8].

The resistance of Gram-negative bacilli against antibiotics is a rapidly rising problem around the globe, Gram-negative bacilli have multiple ways of antibiotic resistance, and they horizontally transfer the resistance genes between species [9]. The antimicrobial resistance issue is particularly shown to be more severe in developing countries, where the infectious disease burden is high and high drug costs prevent the widespread application of newer, more expensive agents [10]. In Sudan, UTIs are responsible for large numbers of outpatient visits and hospital-acquired infections, with the prevalence of multidrug-resistant uropathogens increasing [11]. In 2017 it was reported that more than two-thirds of gram negative isolates causing UTI were resistant in Sudan [12].

There is high variability in the prevalence and types of pathogens that cause UTIs and resistance patterns; these pathogens must be identified to be able to continually update and increase the effectiveness of empirical and targeted therapies [13]. Determining the types of pathogens that cause UTIs and the resistance pattern is essential for formulating and monitoring effective UTI treatment plans. Identification of their resistance pattern of genes is necessary for the surveillance of their transmission in hospitals and to overcome the problems associated with Gram-negative bacilli resistance. This study aims to gather more information regarding pathogens and antimicrobial resistance in Sudan to estimate the spread of multidrug-resistant gram-negative bacilli.

## Materials and methods

This was a descriptive cross-sectional hospital-based study conducted in three hospitals in Khartoum State (El-Amal Hospital, Bahri Teaching Hospital, and Al-Baraha Hospital) from March to October 2022. The study population was patients (inpatients and outpatients) who presented to the selected hospitals and were suspected of having UTI. Patients with an autoimmune disease, HIV patients, and those taking antibiotics in the past 48 h were excluded from the study. Finally, patients who were confirmed by laboratory investigations to have urinary tract infections and yielded culture growth were included.

### Data collection

The researcher used a structured data collection sheet that included patient-related factors like age, gender, diabetes, history of hospitalization, and history of UTI to collect data.

### Urine samples

Samples of midstream urine specimens were collected from suspected patients in a sterile wide-mouth container and then transferred to the laboratory within two hours of collection.

### Bacterial culture and identification

Using a calibrated wire inoculating loop (0.001 ml), urine samples were inoculated onto Cystine Lactose Electrolyte Deficient Medium. Cultures were incubated in an aerobic atmosphere at 37ºC for 24 h. Colonies were counted to check for the presence of significant bacteriuria. A colony count yielding bacterial growth of 10^5^ CFU/ml of urine was regarded as significant bacteriuria (SB) [14]. All positive cultures with significant bacteriuria were then identified at the species level by their colony characteristics, gram-staining reaction, and the pattern of biochemical profiles using standard procedures. The tests used to identify the Enterobacterales [15] included those for indole production, H2S production in KIA agar (Klinger’s iron agar), citrate utilization, motility test, urease test, and carbohydrate utilization (Supplementary 1).

### Antimicrobial susceptibility testing

The antimicrobial susceptibility testing of all identified isolates in urine samples was done according to the Clinical and Laboratory Standards Institute (CLSI) criteria (30th Edition) [16]. Briefly, from a pure culture, a loopful of bacterial colonies was taken and transferred to a tube containing 5 ml of normal saline and mixed gently until they formed a homogenous suspension. The suspension’s turbidity was then adjusted to the density of a McFarland 0.5 to standardize the inoculum size. A sterile cotton swab was gently rotated against the tube’s surface after being dipped into the suspension to remove any surplus. The swab then distributed the bacteria evenly over the entire surface of Mueller-Hinton agar (Oxoid). The inoculated plates were left at room temperature to dry for 3–5 min. Using a clean needle, antibiotic discs of the following concentrations were put on the surface of Mueller-Hinton agar (Oxoid): amikacin (30 μg), cefotaxime (30 μg), ceftazidime (30 μg), ceftriaxone (30 μg), cefixime (5 μg), ciprofloxacin (5 μg), colistin (10 μg), gentamicin (10 μg), imipenem (10 μg), meropenem (10 μg), norfloxacin (10 μg), and trimethoprim-sulfamethoxazole (25 μg) (Supplementary 2), cation-adjusted Mueller–Hinton broth was used for colistin. The criteria used to select the antimicrobial agents tested were based on their availability and frequent prescriptions to manage UTIs in the study area. The plates were then incubated at 37ºC for 24 h. The zone of inhibition around the discs was measured using a digital caliper, and the isolates were classified as sensitive or resistant.

### Investigation of resistant genes by PCR

The isolates that exhibited antibiotic resistance were subjected to PCR (polymerase chain reaction) to detect the presence of the *bla*_TEM_, *bla*_SHV_, and *bla*_CTX-M_ genes. For DNA extraction, the boiling method was applied. Firstly, three to five colonies were picked from fresh culture medium, and then a suspension was prepared using 200 ml of distilled water boiled at 100˚C for 30 min. The suspension was then centrifuged at 12,000 rpm for 30 min, and the supernatant containing DNA was quantified using UV-Vis spectrophotometry and transferred to new Eppendorf tubes for PCR to amplify the genes [17] (*bla*_TEM_, *bla*_SHV_, and *bla*_CTX-M_). To perform PCR, 2 μl of the primers (Supplementary 3), 5 μl of the extracted DNA, and 13 μl of distilled water were added to the PCR Master Mix ((Maxime PCR Permix Kit, Korea 20 μl). Thermal cycling for 30 cycles was done at 94 °C for 1 min, 60 °C for 1 min, and 72 °C for one and a half min. The final extension step was performed for 5 min at 72 °C. The PCR products were applied and electrophoresed in a 2% agarose gel along with ladder DNA, and then stained using ethidium bromide. The result was observed by the transilluminator system (Supplementary 4). DNA from reference *bla*_CTX-M_, *bla*_TEM,_ and *bla*_SHV_-like-positive strains was used as a positive control.

## Results

This study included 50 patients with UTI out of 229 suspected patients (21.8%). The most prominent group of patients was older than 60 years (40%); the majority were females (70%). Only 20% were diabetic, 6% had a hospitalization history, 8% had obstructive urinary disease, and 10% had a history of UTI (Table 1).

**Fig. 1:**
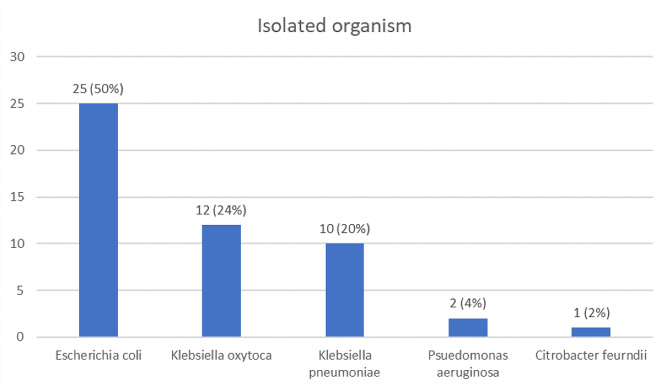
Isolated organisms from urine samples of study participants (*n* = 50)

In culture, *Escherichia coli* was the most common isolated organism (50%), followed by *Klebsiella oxytoca* (24%), *Klebsiella pneumoniae* (20%), *Pseudomonas aeruginosa* (4%), and *Citrobacter freundii* (2%) (Fig. 1).

**Table 1:** Characteristics of patients diagnosed with UTI (*n* = 50)

	Frequency (%)
Age	
18–31	5 (10%)
32–45	10 (20%)
46–59	15 (30%)
> 60	20 (40%)
Gender	
Male	15 (30%)
Female	35 (70%)
Diabetes Mellitus	10 (20%)
History of hospitalization	3 (6%)
Obstructive urinary disease	4 (8%)
History of UTI	5 (10%)

Regarding resistance to antibiotics, organisms showed good susceptibility to colistin (83%), with 17% resistance, while resistance to the rest of the antibiotics was 77% to amikacin. 97.6% to cefotaxime, 96.8% to ceftazidime, 97.6% to ceftriaxone, 96.8 to cefixime, 87.6 to ciprofloxacin, 88.4% to gentamycin, 62% to imipenem, 67.6% to meropenem, 87.6% to norfloxacin, and 95.6% to trimethoprim. All organisms showed the highest susceptibility to colistin: *Escherichia coli* 100%, *Klebsiella oxytoca* 75%, *Klebsiella pneumoniae* 90%, *Pseudomonas aeruginosa* 50%, and *Citrobacter freundii* 100% (Table 2).

The most prevalent genes for resistance were *bla*_TEM_ (100%), *bla*_CTX-M_ (94%), and *bla*_SHV_ (84%) (Table 3).

**Table 2:** Antibiotic susceptibility of the isolated organisms (*n* = 50)

Organism	AK	CTX	CAZ	CRO	CFM	CIP	CT	GN	IPM	MEM	NOR	SXT	Overall
*E. coli*	60%	4%	8%	4%	8%	4%	100%	40%	72%	60%	4%	4%	30%
*K. oxytoca*	25%	8%	8%	8%	8%	8%	75%	8%	58%	42%	8%	8%	22%
*K. pneumoniae*	30%	0	0	0	0	0	90%	10%	10%	10%	0	10%	13%
*P. aeruginosa*	0	0	0	0	0	50%	50%	0	50%	50%	50%	0	20.8%
*C. freundii*	0	0	0	0	0	0	100%	0	0	0	0	0	8.3%
Susceptibility	23%	2.4%	3.2%	2.4%	3.2%	12.4%	83%	11.6%	38%	32.4%	12.4%	4.4%	19%
Resistance	77%	97.6%	96.8%	97.6%	96.8%	87.6%	17%	88.4%	62%	67.6%	87.6%	95.6%	81%

AK: Amikacin, CTX: Cefotaxime, CAZ: Ceftazidime, CRO: Ceftriaxone, CFM: Cefixime, CIP: Ciprofloxacin, CT: Colistin, GN: Gentamicin, IPM: Imipenem, MEM: Meropenem, NOR: Norfoxacin, SXT, Trimethoprim

**Table 3:** Results of the resistance genes in isolated organisms (*n* = 50)

Organism	Number	Bla_TEM_	Bla_SHV_	Bla_CTX−M_
*Escherichia coli*	25	25 (100%)	25 (100%)	24 (96%)
*Klebsiella oxytoca*	12	12 (100%)	9 (75%)	10 (83%)
*Klebsiella pneumonia*	10	10 (100%)	6 (60%)	10 (100%)
*Pseudomonas aeruginosa*	2	2 (100%)	2 (100%)	2 (100%)
*Citrobacter freundii*	1	1 (100%)	0	1 (100%)
Total	50	50 (100%)	42 (84%)	47 (94%)

## Discussion

The effectiveness of antibiotics in treating UTIs is becoming increasingly limited due to the rising resistance of Gram-negative bacilli. Antibiotic resistance poses a significant challenge in the management of UTIs as it reduces the available treatment options, increases the risk of treatment failure, and causes adverse economic consequences, including increased healthcare expenditures brought on by a rise in hospital admissions and drug use [18] along with more morbidity and mortality [19]. This makes it crucial to understand the patterns of antibiotic resistance in UTI-causing bacteria in order to develop effective treatment strategies and prevent the spread of multidrug-resistant strains.

The study included 50 patients who were confirmed to have UTIs and yielded culture growth. The most commonly identified organism was *Escherichia coli*, constituting half of the isolates, while the rest were *Klebsiella species* and, to a lesser extent, *Pseudomonas aeruginosa* and *Citrobacter freundii*. This is similar to what recent studies have reported; as *Escherichia coli* is the most common isolated organism in UTI, as reported in a meta-analysis by Reza et al. (2019) [20]. Likewise, Majumder et al. (2022) in Bangladesh found that *Escherichia coli* was the commonest isolated organism causing UTI followed by *Klebsiella species* [21], Ahmed et al. (2019) also found that *Escherichia coli* was the commonest isolated organism causing UTI followed by *Klebsiella* pneumoniae in Saudi Arabia [22]. In contrast, a recent study in Sudan by El Badawi et al. (2019) reported that *Klebsiella pneumoniae* was the most common organism causing UTI [23].

It was found that isolates were almost resistant to amikacin, cefotaxime, ceftazidime, ceftriaxone, cefixime, ciprofloxacin, gentamycin, imipenem, meropenem, norfloxacin, and trimethoprim. Colistin, on the other hand, worked the best overall, with only 17% overall resistance. However, Majumder et al. (2022) in Bangladesh reported that nitrofurantoin was the most effective antibiotic, despite the fact that the resistance profiles of the organisms causing UTI vary from study to study, but they all share the same pattern of cephalosporine resistance [21]. Reza et al. (2019) [20] reported that antibiotics like imipenem, amikacin, and ciprofloxacin were effective. This difference in results suggests that the resistance patterns of organisms that cause UTIs may change over time and in different places.

In this study the overall resistance for all gram negative isolates was 81%, emphasizing that the antibiotic resistance issue is progressing as in 2017 Saeed et al. reported that 67% of gram negative bacteria causing UTI were resistant [12], and providing a strong alarming signal for to optimize treatment according to the resistance profile and to conduct public interventions to limit the extent of the issue.

The *bla*_TEM_ gene was the most commonly detected gene responsible for the resistance, as it was detected in all isolates; however, *bla*_CTX-M_ and *bla*_SHV_ were also detected in the majority of bacterial isolates. Similarly, in Sudan, Satir et al. (2016) found that the *bla*_TEM_ gene was the commonest gene among resistant isolates, as it was found in more than two-thirds of them [17], and similarly in Saudi Arabia [24]. It is now well known that among *Escherichia coli* and *Klebsiella pneumoniae*, the predominant mechanisms for resistance are ESBLs belonging to the CTX-M, TEM, and SHV families, as reported by Bedenić et al. (2021) [25]. The high prevalence of *bla*_TEM_, *bla*_SHV_, and *bla*_CTX-M_ genes among Gram-negative bacilli is a cause for concern. Extensive surveillance investigations should be done to get a comprehensive picture of the transmission and epidemiology of these isolates bearing resistance genes.

This study is not free of constraints, primarily due to its narrow scope and concentration on only three particular resistance genes. Additional investigation is required to examine supplementary mechanisms of resistance and their genetic underpinnings in order to achieve a broader comprehension.

## Conclusion

The findings of this study indicate that *Escherichia coli* and *Klebsiella species* are the most common uropathogens causing urinary tract infections in Khartoum State. These pathogens also showed high levels of resistance to several commonly used antibiotics, with the *bla*_TEM_, *bla*_SHV_, and *bla*_CTX-M_ genes being the most prevalent resistance genes. The high prevalence of antibiotic resistance in UTI-causing bacteria is a concerning issue, as it limits the available treatment options and increases the risk of treatment failure.

### Electronic supplementary material

Below is the link to the electronic supplementary material.

**Supplementary Material 1:** Biochemical characteristics of bacteria identified in urine samples of UTI diagnosed patients

**Supplementary Material 2:** Antibiotics used for susceptibility testing

**Supplementary Material 3:** Shows primers sequence for the three targeted genes

**Supplementary Material 4:** Agarose gel result of *bla*_TEM_, *bla*_SHV_, and *bla*_CTX−M_ genes

## Data Availability

Data are available upon reasonable request from the corresponding author.

## Abbreviations

AMEs: Aminoglycosides-Modifying Enzymes
CLSI: Clinical and Laboratory Standards Institute
CTX-M: Cefotaximase
ESBL: Extended-Spectrum-Β-Lactamases
HIV: Human Immunodeficiency Virus
KIA: Klinger’s Iron Agar
MGEs: Mobile Genetic Elements
PCR: Polymerase Chain Reaction
SB: Significant Bacteriuria
SHV: Sulfhydryl-Variable
TEM: Temoneira
UTIs: Urinary Tract Infections
