# Supplementary material for: Assessment of BlaTEM, BlaSHV, and BlaCTX-M genes of antibiotic resistance in Gram-negative bacilli causing urinary tract infections in Khartoum State: a cross-sectional study
Source: BMC Infect Dis. 2024 Jan 29;24:141. doi: 10.1186/s12879-024-09023-7 (PMC10826001; doi:10.1186/s12879-024-09023-7)
Supplement: Supplementary file 1 — Supplementary Material 1: Biochemical characteristics of bacteria identified in urine samples of UTI diagnosed patients [file 12879_2024_9023_MOESM1_ESM.docx]

Supplementary 1: Biochemical characteristics of bacteria identified in urine samples of UTI diagnosed patients.

| Organism | Lactose fermentation | Indole | Citrate | Urea | KIA | Motility | H2S | Gas |
| --- | --- | --- | --- | --- | --- | --- | --- | --- |
| *Citrobacter freundii* | + | - | + | - | RY | + | D | - |
| *Escherichia coli* | + | + | - | - | YY | + | - | + |
| *Klebsiella oxytoca* | + | + | + | + | YY | - | - | - |
| *Klebsiella pneumoniae* | + | - | + | + | YY | - | - | - |
| *Pseudomonas aeruginosa* | - | - | + | D | RR | + | - | - |

**Key:**

(+): Means positive reaction

(-): Means negative reaction

D: Means delayed reaction

KIA: Kliger's Iron Agar

RY (red yellow): Means fermentation of lactose and non-fermentation of glucose

YY (yellow yellow): Means fermentation of lactose and glucose

RR (red red): Means non fermentation of lactose and glucose
