# Supplementary material for: Assessment of BlaTEM, BlaSHV, and BlaCTX-M genes of antibiotic resistance in Gram-negative bacilli causing urinary tract infections in Khartoum State: a cross-sectional study
Source: BMC Infect Dis. 2024 Jan 29;24:141. doi: 10.1186/s12879-024-09023-7 (PMC10826001; doi:10.1186/s12879-024-09023-7)
Supplement: Supplementary file 2 — Supplementary Material 2: Antibiotics used for susceptibility testing [file 12879_2024_9023_MOESM2_ESM.docx]

Supplementary 2: Antibiotics used for susceptibility testing.

| Antibiotic | Concentration | Abbreviation | zone of inhibition (mm) |
| --- | --- | --- | --- |
| Amikacin | 30 | AK | 19-26 |
| Cefotaxime | 30 | CTX | 17-22 |
| Ceftazidime | 30 | CAZ | 17-22 |
| Ceftriaxone | 30 | CRO | 29-35 |
| Cefixime | 5 | CFM | 23-27 |
| Ciprofloxacin | 5 | CIP | 30-40 |
| Colistin | 10 | CT | >10 |
| Gentamicin | 10 | GN | 19-26 |
| Imipenem | 10 | IPM | 26-32 |
| Meropenem | 10 | MEM | 28-34 |
| Norfoxacin | 10 | NOR | 28-35 |
| Trimethoprim | 25 | SXT | 23-24 |
