# Supplementary material for: Assessment of BlaTEM, BlaSHV, and BlaCTX-M genes of antibiotic resistance in Gram-negative bacilli causing urinary tract infections in Khartoum State: a cross-sectional study
Source: BMC Infect Dis. 2024 Jan 29;24:141. doi: 10.1186/s12879-024-09023-7 (PMC10826001; doi:10.1186/s12879-024-09023-7)
Supplement: Supplementary file 3 — Supplementary Material 3: Shows primers sequence for the three targeted genes [file 12879_2024_9023_MOESM3_ESM.docx]

Supplementary 3: Shows primers sequence for the three targeted genes.

|  | Sequence (5′- 3′) | Product  Size | Reference |
| --- | --- | --- | --- |
| Gene |  |  |  |
| TEM FP | TCGCCGCATACACTATTCTCAGAATGA | 445 | [17] |
| TEM RP | ACGCTCACCGGCTCCAGATTTAT |  |  |
| SHV FP | ATGCGTTATATTCGCCTTGTG | 747 | [17] |
| SHV FP | TGCTTTGTTATTCGGGCCAA |  |  |
| CTX FP | ATGTGCAGYACCAGTAARGTKATGGC | 593 | [17] |
| CTX FP | GGGTRAARTARGTSACCAGAAYCAGCGG |  |  |
