# Supplementary material for: Assessment of BlaTEM, BlaSHV, and BlaCTX-M genes of antibiotic resistance in Gram-negative bacilli causing urinary tract infections in Khartoum State: a cross-sectional study
Source: BMC Infect Dis. 2024 Jan 29;24:141. doi: 10.1186/s12879-024-09023-7 (PMC10826001; doi:10.1186/s12879-024-09023-7)
Supplement: Supplementary file 4 — Supplementary Material 4: Agarose gel result of blaTEM, blaSHV, and blaCTX−M genes [file 12879_2024_9023_MOESM4_ESM.docx]

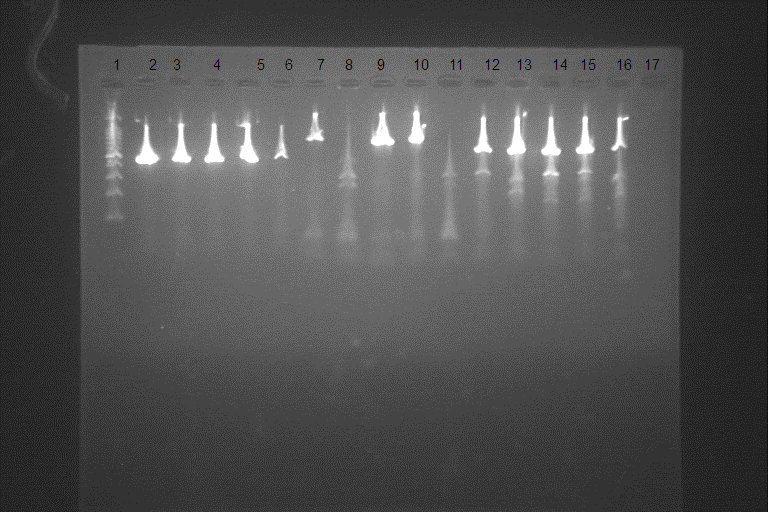


lane: 2,3,4,5,6: are *bla*_TEM_ positive samples.

lane 7,9,10: are *bla*_SHV_ positive samples, lane 8,11: are *bla*_SHV_ negative samples.

lane 12,13,14,15,16: are *bla*_CTX-M_ positive samples.

lane 17: Negative control.

Supplementary 4: Agarose gel result of *bla*_TEM_, *bla*_SHV_, and *bla*_CTX-M_ genes.
